# A Multi-year Beneficial Effect of Seed Priming with Gibberellic Acid-3 (GA_3_) on Plant Growth and Production in a Perennial Grass, *Leymus chinensis*

**DOI:** 10.1038/s41598-018-31471-w

**Published:** 2018-09-04

**Authors:** Hong-Yuan Ma, Dan-Dan Zhao, Qiu-Rui Ning, Ji-Ping Wei, Yang Li, Ming-Ming Wang, Xiao-Long Liu, Chang-Jie Jiang, Zheng-Wei Liang

**Affiliations:** 1 Northeast Institute of Geography and Agroecology, Chinese Academy of Sciences (CAS), Beijing, China; 2 Institute of Agrobiological Sciences, National Agriculture and Food Research Organization (NARO), Tsukuba, Japan

## Abstract

Seed priming is a widely used technique in crops to obtain uniform germination and high-quality seedlings. In this study, we found a long-term effect of seed priming with gibberellic acid-3 (GA_3_) on plant growth and production in *Leymus chinensis*. *S*eeds were germinated on agar plates containing 0–200 μM GA_3_, and the germinated seedlings were transplanted to clay planting pots and grown for about one year. The clonal tillers grown from the mother plants were transplanted to field conditions in the second year. Results showed that GA_3_ treatment significantly increased seed germination rate by 14–27%. GA_3_ treatment also promoted subsequent plant growth and biomass production, as shown by a significant increase in plant height, tiller number, and fresh and dry weight in both pot (2016) and field (2017) conditions. It is particularly noteworthy that the growth-promoting effect of a single seed treatment with GA_3_ lasted for at least two years. In particular, GA_3_ treatment at 50 μM increased aboveground fresh and dry weight by 168.2% and 108.9% in pot-grown conditions, and 64.5% and 126.2% in field-grown conditions, respectively. These results imply a transgenerational transmission mechanism for the GA-priming effect on clonal offspring growth and biomass production in *L. chinensis*.

## Introduction

Seed quality is the basis of adequate plant establishment and is associated with the productive success of crops^1^. Therefore, a variety of strategies are employed in improving seed germination, seedling growth, and productivity. Seed priming, a low-cost and low-risk tool, is considered to be the most effective of these methods^1–4^. It comprises a pre-sowing treatment of soaking seeds in a specified solution, allowing some metabolic activities to proceed before germination^5,6^, and can increase germination percentage, shorten germination time, and improve seedling establishment^7,8^. Based on the priming agents, seed priming can generally be classified into four groups: hydropriming, osmopriming, halopriming, and hormone priming^6^.

In hormone priming, plant growth regulators such as gibberellic acids (GAs)^5^, abscisic acid (ABA)^9,10^, or salicylic acid (SA)^1,11,12^ have been widely used to increase synchronized seed germination, seedling growth, and also the yield of a variety of crop species, such as rice^5,10,13^, corn^14,15^, safflower^16^, wheat^12,17,18^, beet^1^, and sunflower^19^. However, most studies have focused on the life stages of seed germination and seedling growth, and little attention has been given to priming effects over a long-time span. Moreover, there have been scarce reports of studies on hormone priming in grass species, especially perennial grass species^20,21^.

GAs play important roles in many essential plant growth and development processes, including seed germination, stem elongation, leaf expansion, flower and fruit development, and floral transition^22^. They are often used to overcome seed dormancy, and can significantly improve seed germination in many species, mainly through the activation of embryo growth, mobilization of reserves, and weakening of the endosperm layer^3,15^. It has also been reported that seed priming with GAs improves germination and the growth parameters of shoot length, root length, and seedling weight in *Capparis spinosa*^7^, *Trigonella foenum-graecum*^11^, *Hibiscus sabdariffa* L.^9^, *Trifolium repens* L.^20^, *Beta vulgaris*^1^, *Zea mays* L.^14^, and *Medicago sativa*^21^. In addition, seed priming with GAs increased yield in sunflower^12^ by 123.5% (seeds per head) and wheat^19^ by 7.0% (grain yield).

The most important function of plants in grassland ecosystems is their contribution to natural grassland ecosystem productivity, which partially relies on the plant biomass of individual plants, especially perennial species^23–25^. *Leymus chinensis*, a perennial, rhizomatous grass is one of the most widely distributed types of steppe vegetation in temperate eastern Eurasia^26,27^. It is one of the species most preferred for consumption by large herbivores because of its high palatability in terms of forage value, and high crude protein^28^. In recent years, due to human interference, especially overgrazing, *L. chinensis* grassland has become severely degraded, to unprecedented levels^26,27^, and one of the most serious problems facing the development of animal husbandry is now the reduction of plant height and productivity in *L. chinensis*. Therefore, improving the primary productivity of *L. chinensis* in both natural and artificial grassland has become an urgent necessity.

In this study, the objective was to elucidate the priming effects of gibberellic acid-3 (GA_3_) on seed germination and subsequent growth and grass production in the perennial grass, *L. chinensis*.

## Results

### Effect of GA_3_ treatment on seed germination

**Figure 1:**
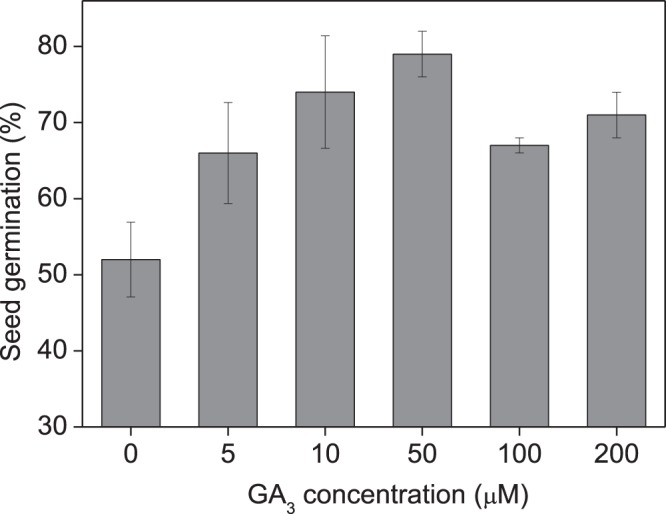
Effect of the application of gibberellic acid-3 (GA_3_) on seed germination in *Leymus chinensis*. Seeds were germinated in agar plates containing 0 (control), 5, 10, 50, 100, and 200 μM of GA_3_, for 28 d under an alternating cycle of 12/12 h of light (fluorescent and incandescent white light of 54 μmol m^−2^ s^−1^) at 28/16 °C. Values are mean ± s.e.

GA_3_ treatment at all concentration levels significantly enhanced seed germination rates compared with the control treatment, with the highest recorded germination rate being 50 μM (Fig. 1). The effect of GA_3_ increased with increasing concentration up to 50 μM, while further higher concentrations (100 and 200 μM) slightly compromised the promoting effect when compared to concentrations of 10 and 50 μM. GLM analysis showed that GA_3_ concentrations had significant promoting effects (df = 5, χ^2^ = 19.383, p < 0.01), and the Tukey’s test showed that a concentration of 50 μM was the most effective in promoting seed germination, when compared to the control (p < 0.001).

### Effect of seed priming with GA_3_ treatment on growth in pot experiments (2016)

**Figure 2:**
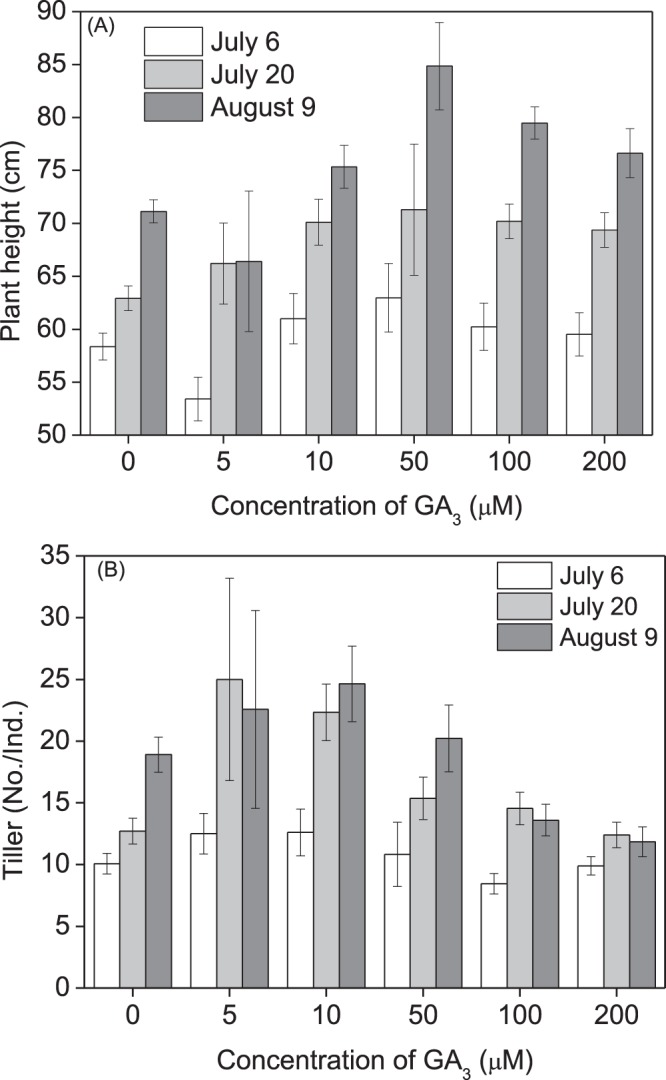
Effect of seed priming with gibberellic acid-3 (GA_3_) on plant growth in *Leymus chinensis* grown in pots (2016). *Leymus chinensis* seeds were germinated in the presence of GA_3_ at different concentrations, as indicated (0–200 μM), and the seedlings were transplanted to and grown in clay pots. (**A**) Plant height, (**B**) tillers per plant. Columns in white, light grey, and grey represent the measurements made on July 6, July 20, and August 9, 2017, respectively. Data are means ± s.e.

Seed treatment with GA_3_ at all concentration levels promoted subsequent plant height (df = 5, F = 5.704, p < 0.001) and tiller number (df = 5, F = 8.360, p < 0.001) during the whole growth period (Fig. 2). Plant height was highest at 50 μM, and tiller number was greatest at 5 μM and 10 μM GA_3_, respectively (Fig. 2).

**Figure 3:**
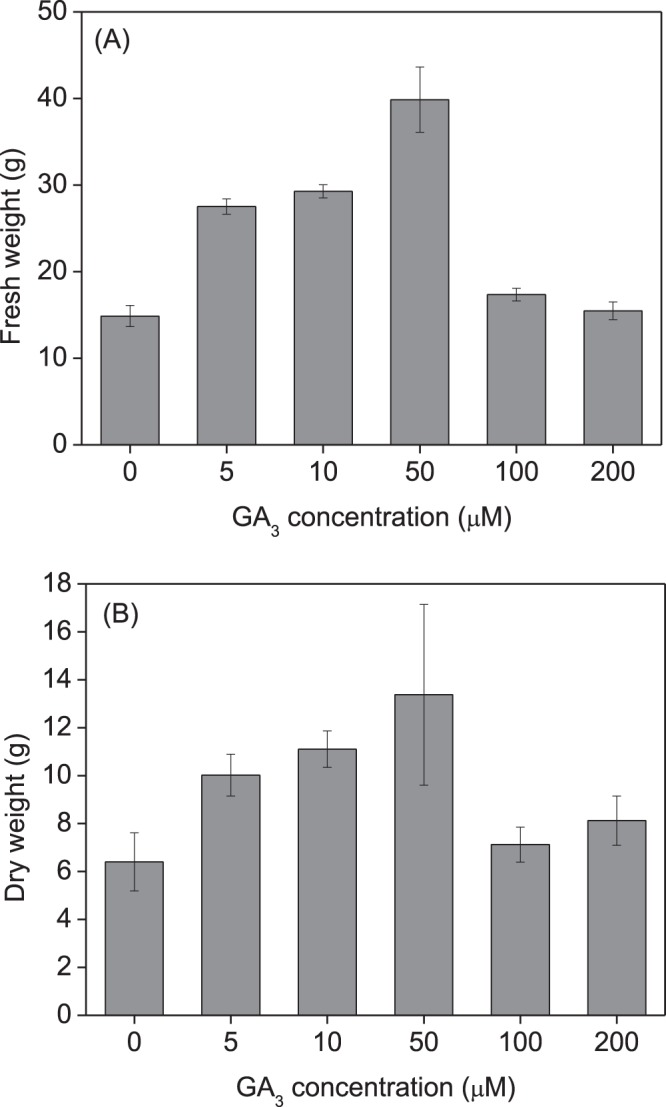
Effect of seed priming with gibberellic acid-3 (GA_3_) on fresh and dry weight of *Leymus chinensis* grown in pots (2016). *Leymus chinensis* seeds were germinated in the presence of GA_3_ at different concentrations, as indicated (0–200 μM), and the seedlings were transplanted to and grown in clay pots. (**A**) Fresh weight, (**B**) dry weight per plant. Data are means ± s.e.

Grass production was also markedly enhanced by seed treatment with GA_3_ (Fig. 3). Both the fresh (df = 5, F = 4.570, p = 0.017) and dry weight (df = 5, F = 4.428, p = 0.019) of shoots were significantly affected by GA_3_ concentrations, with a GA_3_ treatment of 50 μM showing the highest promoting effect. No significant effect on grass production was observed when GA_3_ concentrations was ≥100 μM (Fig. 3).

### Transgenerational effects of seed priming with GA_3_ treatment on clonal offspring growth in field conditions (2017)

**Figure 4:**
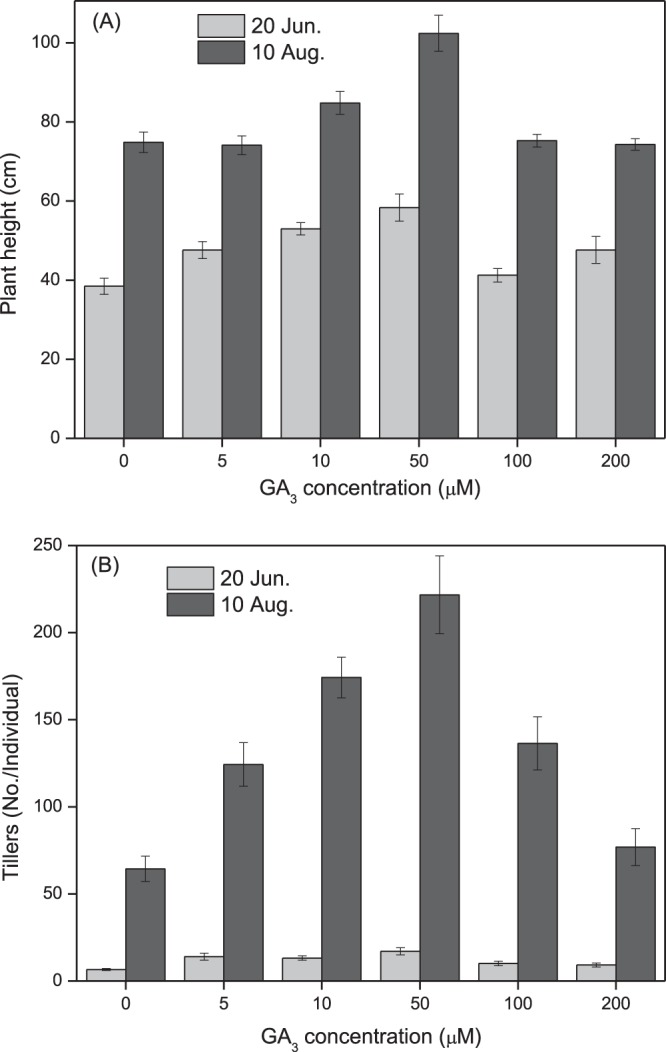
Effect of seed priming with gibberellic acid-3 (GA_3_) on plant growth of *Leymus chinensis* in the field experiment (2017). *Leymus chinensis* seeds were germinated in the presence of GA_3_ at different concentrations, as indicated (0–200 μM), and the seedlings were transplanted to and grown in clay pots in 2016. The tillers grown from the mother plants were separated individually and transplanted to the field in 2017. (**A**) Plant height, (**B**) tillers per plant. The columns in light grey and grey represent the measurements made on July 20 and August 10, 2017, respectively. Data are means ± s.e.

Seed treatment with GA_3_ at all concentration levels promoted clonal offspring plant growth during the whole growth period, as shown by the increased plant height and tiller number per plant (Fig. 4). Values of plant height and tiller number were highest at 50 μM GA_3_ (Fig. 4A). Plant height (df = 5, F = 19.458, p < 0.001) of *L. chinensis* was significantly affected by GA_3_ treatment. Significant promotion of tiller number by GA_3_ concentration (df = 5, F = 11.083, p < 0.001) was observed, especially at a concentration of 50 μM GA_3_ (Fig. 4B).

**Figure 5:**
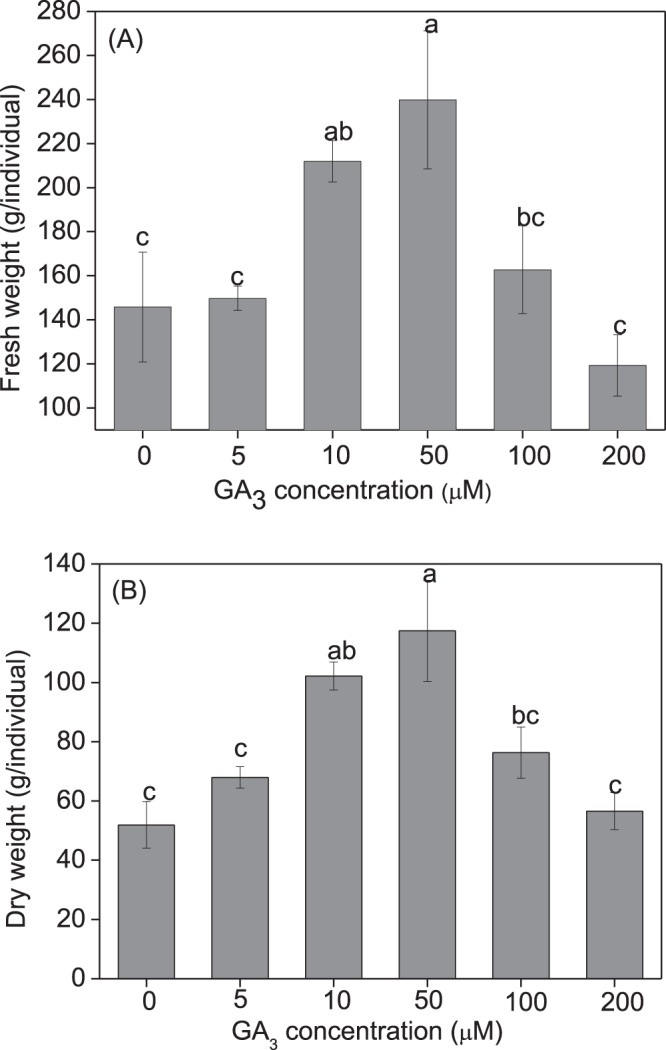
Effect of seed priming with gibberellic acid-3 (GA_3_) on fresh and dry weight of *Leymus chinensis* in the field experiment (2017). *Leymus chinensis* seeds were germinated in the presence of GA_3_ at different concentrations, as indicated (0–200 μM). The seedlings were transplanted to and grown in clay pots in 2016. The tillers grown from the mother plants were separated individually and transplanted to the field in 2017. (**A**) Plant height, (**B**) tillers per plant. The columns in light grey and grey represent the measurements made on July 20 and August 10, 2017, respectively. Data are means ± s.e.

**Figure 6:**
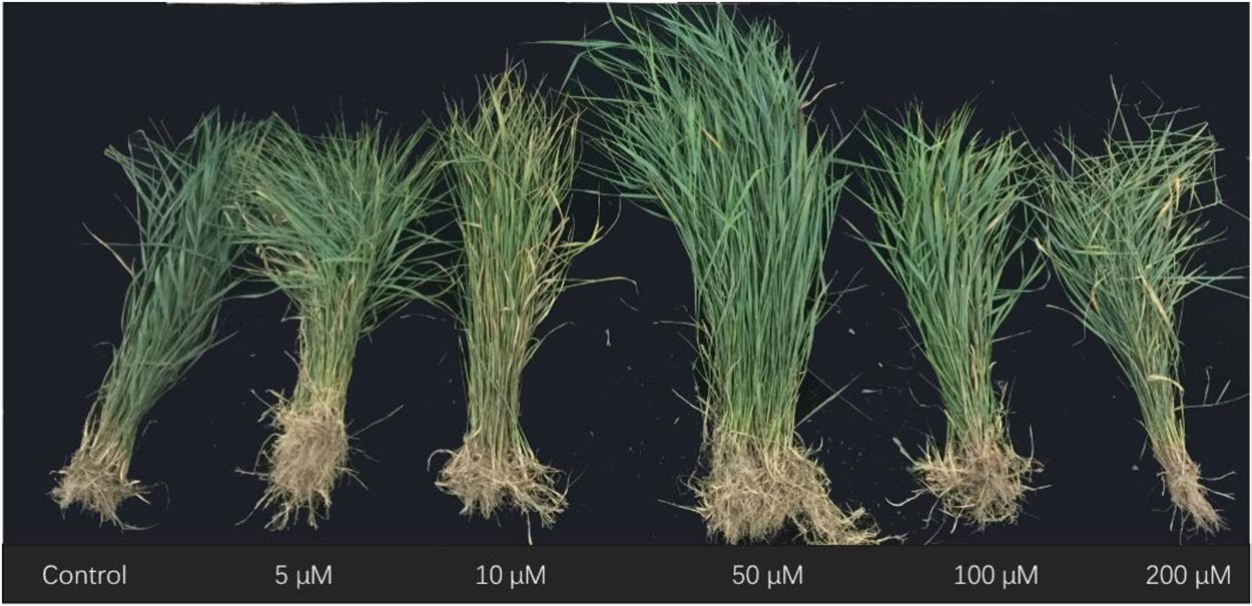
Image of the effect of priming with gibberellic acid-3 (GA_3_) on plant growth in transgenerational *Leymus chinensis* offspring in field conditions (2017).

Grass production was also markedly enhanced by seed treatment with GA_3_ (Figs 5, 6). Both fresh (df = 5, F = 5.279, p = 0.021) and dry (df = 5, F = 8.552, p < 0.001) shoot weights were significantly affected by GA_3_ concentrations, with the 50 μM GA_3_ treatment having the highest promoting effect.

## Discussion

Native perennial species in natural grassland plays a very important role in the broad-scale restoration of degraded ecosystems, where grass reseeding technology has great potential for restoring ecosystem functionality^29,30^. *Leymus chinensis* previously dominated native perennial grass species on the eastern Eurasian Steppe and is considered to be the most attractive grass in the restoration of artificially established grasslands. In this study, seed priming with GA_3_ significantly enhanced seed germination and subsequent plant growth (Figs 2, 4), and grass production (Figs 3, 5) in *L. chinensis*. In particular, GA_3_ priming at a concentration of 50 μM enhanced germination rate by 27.0%, and grass production in fresh and dry matter by 168.2% and 108.9% in pot (Fig. 3), and 64.5% and 126.2% in field (Fig. 5) conditions, respectively. It is noteworthy that the significant improvement in grass production for at least two years was obtained by just a single GA_3_ seed treatment (priming). These results strongly demonstrated that seed priming with GA_3_ is a simple but effective method for enhancing grass production in *L. chinensis*, especially in artificial grasslands where seeding is necessary.

The poor seed germination of *L. chinensis* has been considered an obstacle to the establishment of artificial grasslands^28^. Several strategies for improving seed germination have been suggested, for example, cold stratification, removal of glumes^31^, and exogenous hormone treatments^28^. Seed priming with GA_3_ has been demonstrated to be a useful tool for activating metabolic germination processes and facilitating increments in physiological processes during seed germination^1,4,7^, especially for grass seeds exhibiting physiological dormancy (PD)^3^, e.g., *Leymus arenarius*^32^, *Setaria viridis*^33^, *Tripsacum dactyloides*^34^, and some *Triodia* species (Poaceae)^35^. In *L. chinensis*, we previously proved a positive relationship (p > 0.05) between seed germination and endogenous hormone content during seed development^36^. In this study, exogenous GA_3_ treatment at a range of 5–200 μM enhanced germination rate, with the highest effect recorded at 50 μM (Fig. 1). High GA_3_ concentrations of ≥100 μM showed a less beneficial effect on seed germination compared to concentrations of 10–50 μM. These results are somewhat inconsistent with previous reports that GA_3_ concentrations as high as 300 μM^37^ or 2.89 mM^38^ showed higher promoting effects than other concentrations in *L. chinensis* seed germination. This discrepancy may be ascribed to different degrees of dormancy in the seed material used in the experiments.

GA-priming has been demonstrated to promote seedling growth in various crop plants^4,6^; Seed priming using GA_3_ at appropriate concentrations leads to high germination rates and better seedling growth; however, the beneficial concentration differs among plant species. GA_3_ treatment showed the highest promoting effect on seed germination and seedling growth in *Capparis spinosa* at 360.9 μM^7^, *Trigonella foenum-graecum* at 180.4 μM^11^, and 721.8–1443.5 μM for *Parthenium argentatum* Gray^39^. The yield attributes of *Helianthus annuus* L.^19^ and *Triticum aestivum* L.^12^ were also increased by seed treatment with 10–100 μM GA_3_ for 8 h. In previous studies on *L. chinensis*, GA spraying at various growth stages remarkably promoted plant growth and grass production^40–42^. In this study, we showed that seed priming with GA_3_ significantly promoted plant growth (Figs 2, 4) and enhanced grass production (Figs 3, 5) in *L. chinensis*, in both pot and field experiments. Similar to the effect on seed germination (Fig. 1), seed treatment with GA_3_ at 50 μM yielded the highest promoting effect on plant growth (Figs 2–6), and GA_3_ levels above 50 μM were less beneficial to plant growth than in the range of 5–50 μM (Figs 2–6). This is in accordance with observations that phytohormones only function within a threshold range of concentration levels. However, the most promoting effects of GA_3_ concentrations on production of the first, second, and following generations in *L. chinensis* needs further study.

The most significant and unexpected finding in this study was that the beneficial effect of seed priming with GA_3_ was passed on to clonal offspring for at least two years in *L. chinensis* (Figs 2–6). The fact that the priming effect was also observed in the next generation plants (Figs 4–6) implies a transgenerational transmission mechanism for GA-priming effects in this species. Transgenerational effects have been observed in many species in passing on to offspring maternal stress responses including responses to drought^43^, salinity^44^, and light^45,46^. Hartmann *et al*.^45^ report that far-red irradiated seeds of *Chenopodium album* and *Stellaria media* showed a significantly reduced emergence for two years, demonstrating the influence of the maternal far-red-absorbing seed phytochrome B_fr_ over time^45^. Very recently, Ren *et al*.^47^ have reported that long-term overgrazing-induced memory decreased the photosynthesis of clonal offspring in *L. chinensis* by decreasing leaf chlorophyll content and Rubisco enzyme activity, and downregulating a series of key genes that regulate photosynthetic efficiency, stomata opening, and chloroplast development^47^. It would be very interesting to observe further through how many generations GA priming effects can succeed. The molecular and physiological mechanisms underlying the transgenerational effects of GA-priming remain to be explored, although DNA methylation changes induced by environmental cues have been implicated in many studies of transgenerational effects^43,48^.

## Conclusion

In this study, we showed that seed priming with GA_3_ can significantly enhance seed germination rate and subsequent plant growth and grass production in a perennial grass species, *L. chinensis*. The GA priming effect was transgenerational, with the clonal offspring also showing enhanced plant growth and grass production. Our findings provide a new practical method for improving perennial grass productivity, especially in artificial grasslands, in which seeding is necessary. On the other hand, some questions remain unresolved, such as how long or through how many generations can the GA-priming effect be preserved, and what molecular and physiological mechanisms underpin the transgenerational transmission of GA-priming effects to clonal offspring.

## Materials and Methods

**Figure 7:**
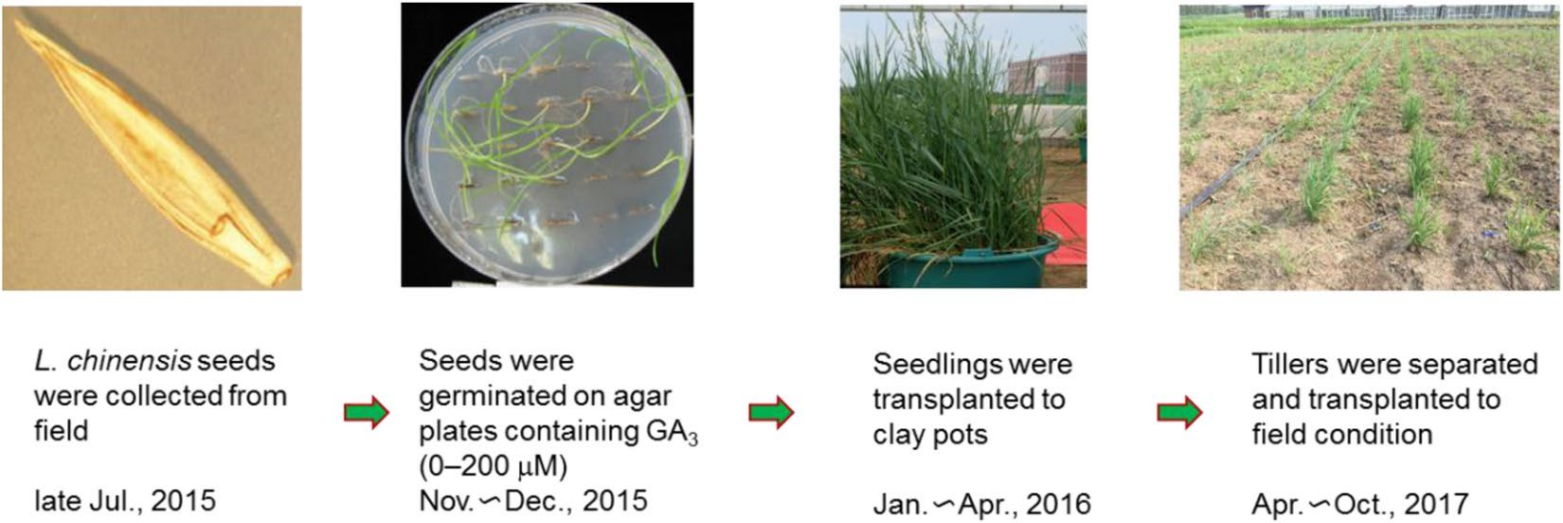
Schematic of the overall experimental design.

A schematic of the overall experimental design is shown in Fig. 7.

### Plant seeds

Matured seeds of *L. chinensis* were collected from the Da’an Sodic Land Experiment Station (45°35′58″–45°36′28″N, 123°50′27″–123°51′31″E), in the western part of the Songnen Plain, northeast China, in late July, 2015. The collected seeds were air-dried at room temperature, placed in a paper bag, and stored at 4 °C until November 2015, just before the germination experiments were undertaken.

### GA_3_ treatment and seed germination (2015)

A 10 mM GA_3_ stock solution was prepared by adding 0.0346 g GA_3_ to a 10 ml volumetric flask and dissolved by a drop of 95% ethylalcohol, after which distilled water was added to make a total solution of 10 ml. Seeds of *L. chinensis* were surface-sterilized in 0.1% HgCl_2_ for 10 min and then washed with distilled water several times before being used in the experiments. Approximately 25 seeds were sown in a Petri dish (diameter 9 cm) containing 0.7% (w/v) water agar supplemented with 0 (control, ethylalcohol at a concentration comparable to that in GA solutions), or 5, 10, 50, 100, 200 μM of GA_3_, with four replicates for each treatment. The Petri dishes were incubated under an alternating cycle of 12/12 h light (fluorescent and incandescent white light of 54 μmol·m^−2^ s^−1^) at 28/16 °C. Seed germination was measured every second day until no new germination occurred within three days.

### Plant growth in pot experiments (2016)

After determining the germination rate, seedlings were transplanted to clay pots (diameter 30 cm, and height 30 cm) each containing 10 kg of soil, in early January 2016. The clay loam soil was collected from a field based at the Northeast Institute of Geography and Agroecology, Chinese Academy of Sciences, Changchun, China. Four seedlings were transplanted into each pot, with four replicates per treatment. Tap water was added to the pots to keep the soil moist. Plant height and tillers per plant were recorded during growth, and grass production (fresh and dry weight of shoots) was measured in late July. The aboveground *L. chinensis* plant material in each pot were cut separately and the fresh weight recorded immediately. Then, the plants were put in paper bags and dried in an oven at 105 °C for 2 h and 80 °C for another 48 h, and the dry weight of each treatment was recorded.

### Plant growth in field experiments (2017)

The *L. chinensis* tillers were separated individually from the mother plants grown in pots, and transplanted to a field with one individual clonal offspring in each hill at the Institute of Geography and Agroecology on April 24, 2017; the field was the same as that from which soil was collected for the pot experiment, and therefore the soil was of the same type. The row and line spacings were 60 cm and 50 cm, respectively. The plot was irrigated once, immediately after the transplantation. Height and tiller number of plants in each hill were recorded on June 20 and August 10, 2017, respectively, with 30 hills for each GA_3_ concentration. Grass production (fresh and dry weight of shoots) was measured in late August, 2017 with 10 replicates for each GA_3_ concentration treatments.

### Statistical analysis

Generalized linear models (GLMs) with a binomial error structure and logit link function were used to compare proportional data relating to the final germination of *L. chinensis* in the GA_3_ treatments. The differences in the tiller number and plant height among GA_3_ treatments were analyzed by repeated measures ANOVA using a linear mixed effect model in the lme4 package in R. We treated sampling time as a random effect and allowing concentration to enter the model as a fixed effect. If necessary, data were log transformed to meet assumptions of normality and homogeneity of variance. In addition, data of fresh and dry weights among different treatments were compared separately by one-way analysis of variance (ANOVA). Before the analyses of ANOVA, the normality (shapiro-wilk test) and homoscedasticity (Levene’s test) was conducted. A Tukey’s test was used for multiple comparisons when the among treatments was significant. All of the analyses were carried out using the R statistical platform^49^.
